# Croatian mayflies (Insecta, Ephemeroptera): species diversity and distribution patterns

**DOI:** 10.3897/zookeys.523.6100

**Published:** 2015-09-28

**Authors:** Marina Vilenica, Jean-Luc Gattolliat, Zlatko Mihaljević, Michel Sartori

**Affiliations:** 1 University of Zagreb, Faculty of Teacher Education, Petrinja, Croatia; 2 Museum of Zoology, Lausanne, Switzerland; 3 University of Zagreb, Faculty of Science, Department of Biology, Division of Zoology, Zagreb, Croatia

**Keywords:** Ephemeroptera, species list, biodiversity, Balkan Peninsula

## Abstract

Knowledge of the mayfly biodiversity in the Balkan Peninsula is still far from complete. Compared to the neighbouring countries, the mayfly fauna in Croatia is very poorly known. Situated at the crossroads of central and Mediterranean Europe and the Balkan Peninsula, Croatia is divided into two ecoregions: Dinaric western Balkan and Pannonian lowland. Mayflies were sampled between 2003 and 2013 at 171 sites, and a total of 66 species was recorded. Combined with the literature data, the Croatian mayfly fauna reached a total of 79 taxa. Of these, 29 species were recorded for the first time in Croatia while 15 species were not previously recorded in Dinaric western Balkan ecoregion. Based on the mayfly assemblage, sampling sites were first structured by ecoregion and then by habitat type. In comparison with the surrounding countries, the Croatian mayfly fauna is the most similar to the Hungarian and Bosnian fauna. Some morphologically interesting taxa such as Baetis cf. nubecularis Eaton, 1898 and *Rhithrogena* from the *diaphana* group were recorded. Ephemera cf. parnassiana Demoulin, 1958, the species previously recorded only from Greece, was also recorded.

## Introduction

Mayflies (Ephemeroptera) have a worldwide distribution, being absent only from Arctic region, Antarctica and some remote oceanic islands (Barber-James et al. 2008). According to the literature (Bauernfeind and Soldán 2012), 369 species are recorded for Europe and North Africa. Mayflies are a merolimnic insect order (i.e. with aquatic larval stages and terrestrial adults) that plays a critical role in running and standing waters where they hold an important position in secondary production, as an important food source for diverse freshwater and terrestrial predators. In recent decades, human impacts on the distribution and abundance of many aquatic insects, including mayflies, are becoming more and more evident. During the 20th century, increasing industrialisation, population growth, overexploitation of natural resources and different types of pollutions have greatly impacted many European freshwater ecosystems, and also endangering the species inhabiting them (Brittain and Sartori 2009). Highly sensitive, confronted with habitat alteration, mayfly species are among the first to disappear. Therefore they are important indicators of freshwater health and widely used in bio-monitoring programmes over the world (Elliott et al. 1988, Sartori and Brittain 2015). The knowledge of the mayfly biodiversity in the Balkan Peninsula is still far from complete. Moreover, many taxa lack appropriate morphological descriptions for the larval and/or adult stages. The mayfly fauna in Croatia is no exception. Published data on Croatian mayflies are generally part of diverse limnological studies (e.g. Matoničkin 1959, 1987, Matoničkin and Pavletić 1961, 1967, Filipović 1976, Habdija and Primc 1987, Habdija et al. 1994, 2004) in which mayflies were investigated only as part of the overall macroinvertebrate fauna. In most studies, identification tools are generally not cited, thus the accuracy of mayfly species identification is questionable. In summary, 50 mayfly species were recorded from Croatia (Bauernfeind and Soldán 2012, Kovács and Murányi 2013, Ćuk et al 2015). In comparison with the number of species recorded in the neighbouring countries, i.e. 68 in Slovenia, 106 in Italy, and 93 in Hungary (Bauernfeind and Soldán 2012), it can be assumed that the Croatian mayfly fauna has been underestimated to date.

Studies on distribution and biodiversity are of crucial importance in determining the conservation status of certain species and in investigating factors that influence that diversity (de Silva and Medellín 2001). Therefore, knowledge of the mayfly faunal composition, seasonal dynamics, distribution, ecology, biogeography and especially their sensitivity as bio-indicators can enable high-quality classification and protection of Croatian freshwater habitats.

## Materials and methods

This research is based on recent mayfly studies conducted in the last decade (2003–2013). The results of field studies were then combined with the literature data given in Bauernfeind and Soldán (2012), Kovács and Murányi (2013) and Ćuk et al. (2015), for the purpose of obtaining a comprehensive checklist of the Croatian mayfly fauna.

### Sampling and laboratory methods

**Figure 1.**
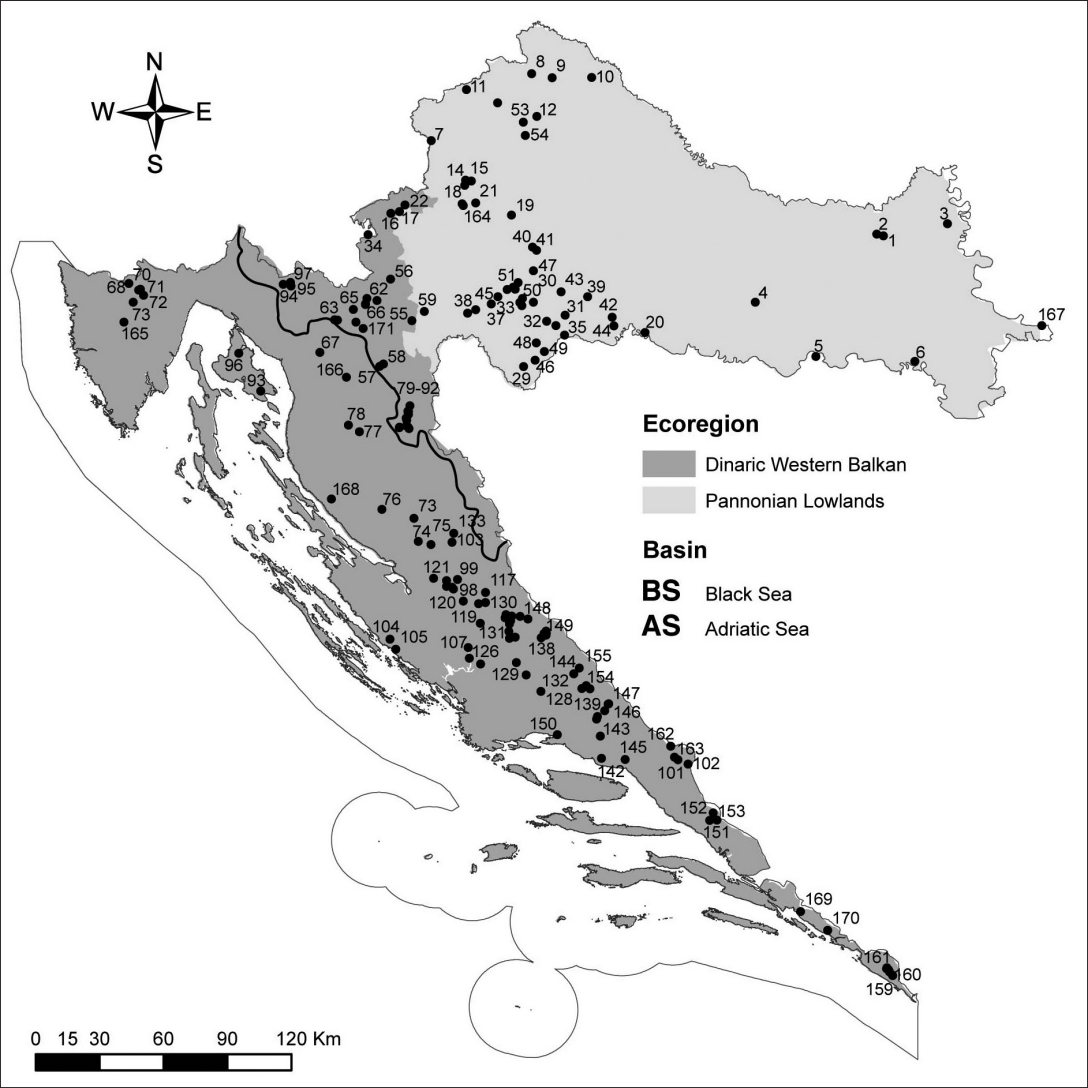
Map of the mayfly fauna sampling sites, Croatia (See Table 1 for codes).

Croatia is a relatively small country situated at the crossroads of Central and Mediterranean Europe and Balkan Peninsula, and is divided into two ecoregions: Dinaric western Balkan (ER5) and Pannonian lowland (ER11) (Illies 1978). Specimens were collected in lotic and lentic freshwater habitats throughout the Croatian territory (Fig. 1). Additionally, specimens housed in the collection of the Slovene National History Museum were identified.

The list of the 171 sampling site names with number codes (site ID), altitude, latitude and longitude is presented in Table 1 as well as on the map (Fig. 1). Larvae were sampled using a Surber sampler and hand net, adults using hand nets and pyramidal emergence traps.

**Table 1.** The list of the sampling sites in Croatia. Ecoregions are taken from Illies (1978); Dinaric western Balkan (5) and Pannonian lowland (11). BS = Black Sea Basin; AS = Adriatic Sea Basin.

Site ID	Sampling site	Altitude	Longitude	Latitude	Ecoregion	Basin
1	Karašica River, Valpovo	85	N45°37'44"	E18°27'28"	11	BS
2	Vučica River, Valpovo	85	N45°38'14"	E18°25'09"	11	BS
3	Čarna channel, Tikveš, near Bilje	85	N45°40'23"	E18°50'46"	11	BS
4	Veličanka River, Mihaljevci	155	N45°21'36"	E17°40'54"	11	BS
5	Sava River, Slavonski Brod	85	N45°07'35"	E18°02'18"	11	BS
6	Sava River, Štitar	80	N45°05'47"	E18°37'38"	11	BS
7	Sutla River, Klanjec	160	N46°02'46"	E15°43'49"	11	BS
8*	Drava River, Varaždin	170	N46°19'50"	E16°20'22"	11	BS
9	Drava River, Čakovec, left drainage ditch	165	N46°18'49"	E16°27'49"	11	BS
10	Drava River, Dubrava, right drainage ditch	145	N46°18'54"	E16°42'15"	11	BS
11	Stream, Trakošćan	275	N46°15'44"	E15°56'30"	11	BS
12	Stiper stream, Ljubešćica, Kalnik Mountain	185	N46°09'04"	E16°22'18"	11	BS
13	Bliznec stream, Medvednica Mountain	380	N45°52'38"	E15°58'33"	11	BS
14	Veliki potok stream, Medvednica Mountain, Mikulići	300	N45°51'29"	E15°56'08"	11	BS
15	Kraljevec stream, Medvednica Mountain	565	N45°52'48"	E15°56'28"	11	BS
16	Sitnik spring, Žumberak-Samoborsko Gorje Mountain	745	N45°44'40"	E15°32'39"	11	BS
17	Slapnica stream, Žumberak-Samoborsko Gorje Mountain	290	N45°44'12"	E15°29'29"	11	BS
18*	Kupa River, Sisak	90	N45°28'32"	E16°22'37"	11	BS
19	Sava River, Rugvica	100	N45°44'01"	E16°13'11"	11	BS
20	Sava River, Mlaka	90	N45°14'14"	E17°01'11"	11	BS
21	Sava River, Zagreb, bridge	110	N45°47'03"	E16°00'10"	11	BS
22	Bregana River, Jarušje	560	N45°46'21"	E15°34'36"	11	BS
23	Stream, Mečenčani	180	N45°17'07"	E16°25'53"	11	BS
24	Stream Zeleni dol, Hrastovica/Hrvatski Čuntić	160	N45°21'51"	E16°16'15"	11	BS
25	Pond Zeleni dol, Hrastovica/Hrvatski Čuntić	160	N45°21'51"	E16°16'18"	11	
26	Petrinjčica River, Prnjavor Čuntićki	150	N45°21'05"	E16°16'57"	11	BS
27	Petrinjčica River, Tješnjak, bridge	150	N45°22'52"	E16°17'11"	11	BS
28	Utinja River, Križ Hrastovački	140	N45°25'15"	E16°14'32"	11	BS
29	Žirovnica River, Donja Ljubina	135	N45°05'39"	E16°17'39"	11	BS
30	Moštanica stream, Moštanica	155	N45°21'55"	E16°21'06"	11	BS
31	Sunja River, Rakovac	120	N45°18'40"	E16°32'33"	11	BS
32	Sunja River, Donji Kukuruzari	150	N45°16'01"	E16°29'14"	11	BS
33	Kupa River, Brest	90	N45°26'56"	E16°15'38"	11	BS
34	Kupa River, Bubnjarci	135	N45°38'42"	E15°21'24"	5	BS
35	Una River, Hrvatska Kostajnica	105	N45°13'37"	E16°32'22"	11	BS
36	Glina River, Marinbrod	100	N45°23'19"	E16°08'20"	11	BS
37	Glina River, Cerjak	110	N45°21'27"	E16°04'58"	11	BS
38	Čemernica stream, Topusko	125	N45°19'08"	E15°57'30"	11	BS
39	Sava River oxbow, Mužilovčica	90	N45°23'23"	E16°40'37"	11	BS
40*	Sava River, Martinska Ves	95	N45°35'09"	E16°22'14"	11	BS
41*	Sava River, Desno Trebarjevo	95	N45°35'56"	E16°20'43"	11	BS
42*	Sava River, Krapje	90	N45°18'10"	E16°49'23"	11	BS
43	Sava River, Lukavec Posavski	90	N45°24'36"	E16°31'03"	11	BS
44	Sava River, Drenov bok	90	N45°15'58"	E16°50'04"	11	BS
45	Mire Plavnica, Šatornja	125	N45°19'58	E16°00'26"	11	
46	Javošnica stream, Donji Javoranj	140	N45°07'14"	E16°21'44"	11	BS
47	Odra River, Sisak	95	N45°29'54"	E16°21'04"	11	BS
48	Zrinčica River, Zrin	240	N45°11'41"	E16°22'13"	11	BS
49	Čatlan River, Gornja Oraovica	170	N45°09'26"	E16°25'03"	11	BS
50	Spring Izvor bijele stijene Križ, Župić	135	N45°25'44"	E16°13'52"	11	BS
51	Šanja River, Gora	140	N45°25'08"	E16°11'42"	11	BS
52	Radonja River, Vojnić	140	N45°19'26"	E15°41'55"	11	BS
53	Lonja River, Brežnički Hum	200	N46°07'34"	E16°17'18"	11	BS
54	Lonja River, Breznica	180	N46°04'11"	E16°18'07"	11	BS
55	Mrežnica River, Generalski stol	140	N45°22'05"	E15°24'55"	5	BS
56	Mrežnica River, Duga Resa	120	N45°27'31"	E15°29'38"	5	BS
57	Dretulja River, Plaški, spring	390	N45°04'31"	E15°20'32"	5	BS
58	Dretulja River, Plaški, middle reach	375	N45°05'06"	E15°21'56"	5	BS
59	Trupinjska rijeka River, Keserov potok	150	N45°17'04"	E15°37'28’	5	BS
60*	Gojačka Dobra River, Gorinci, downstream from the waterfall	160	N45°21'10"	E15°20'44"	5	BS
61*	Gojačka Dobra River, Gorinci, waterfall above the dam	160	N45°20'60"	E15°20'45"	5	BS
62*	Gojačka Dobra River, Tomašići	145	N45°22'33"	E15°21'18"	5	BS
63	Bukovska Dobra River, Turkovići	340	N45°16'59"	E15°10'49"	5	BS
64	Ribnjak stream, Trošmarija	195	N45°19'43"	E15°16'25"	5	BS
65	Vitunjčica stream, Vitunj	340	N45°17'01"	E15°09'48"	5	BS
66	Bistrica stream, Bistrac	230	N45°16'27"	E15°17'28"	5	BS
67	Sušik stream, Drežnica	465	N45°08'44"	E15°04'41"	5	BS
68	Bračana stream, Škuljari	45	N45°24'57"	E13°55'36"	5	AS
69	Rečica stream, Pengari	90	N45°23'21"	E13°59'13"	5	AS
70	Draga River, Selca	160	N45°23'36"	E13°59'46"	5	AS
71	Račićki potok stream, Juradi	50	N45°20'17"	E13°57'20"	5	AS
72	Mirna River, Kotli	155	N45°22'06"	E14°01’	5	AS
73	Jadova River, Gornja Ploča	610	N44°27'03"	E15°38'58"	5	AS
74	Obsenica stream, near Lovinac	560	N44°21'09"	E15°40'36"	5	AS
75	Ričica stream, Ričice	560	N44°20'23"	E15°45'08"	5	AS
76	Lika River, Lički Ribnik	565	N44°29'13"	E15°27'38"	5	AS
77	Gacka River, Ličko Lešće	450	N44°48'46"	E15°19'18"	5	AS
78	Gacka River,Prozor	450	N44°50'23"	E15°15'21"	5	AS
79*	Bijela rijeka River, NP Plitvice Lakes, upper reach	715	N44°50'04"	E15°33'33"	5	BS
80*	Bijela rijeka River, NP Plitvice Lakes, spring	760	N44°49'56"	E15°33'22"	5	BS
81*	Crna rijeka River, NP Plitvice Lakes, spring	710	N44°49'43"	E15°36'49"	5	BS
82*	Crna rijeka River, NP Plitvice Lakes, upper reach	680	N44°50'10"	E15°36'30"	5	BS
83*	Crna rijeka River, NP Plitvice Lakes, lower reach	670	N44°50'22"	E15°35'59"	5	BS
84*	Korana River, NP Plitvice Lakes	390	N44°55'33"	E15°37'09"	5	BS
85*	Plitvica stream, NP Plitvice Lakes	555	N44°54'08"	E15°36'27"	5	BS
86*	Tufa barrier Novakovića Brod, NP Plitvice Lakes	510	N44°54'07"	E15°36'38"	5	BS
87*	Tufa barrier Labudovac, NP Plitvice Lakes	630	N44°52'17"	E15°35'59"	5	BS
88*	Tufa barrier Kozjak-Milanovac, NP Plitvice Lakes	545	N44°53'39"	E15°36'32"	5	BS
89*	Kozjak Lake, NP Plitvice Lakes	555	N44°53'18"	E15°36'38"	5	BS
90*	Prošće Lake, NP Plitvice Lakes	665	N44°51'51"	E15°36'06"	5	BS
91*	Ciginovac Lake, NP Plitvice Lakes	640	N44°52'22"	E15°35'51"	5	BS
92*	Kaluđerovac Lake, NP Plitvice Lakes	540	N44°54'05"	E15°36'41"	5	BS
93	Suha Ričina stream, Jurandvor, Krk island	20	N44°58'38"	E14°43'52"	5	AS
94	Zeleni vir, Skrad	540	N45°25'25"	E14°53'53"	5	BS
95	Curak stream, Zeleni vir	330	N45°25'37"	E14°53'33"	5	BS
96	Veli potok stream, Dobrinj, Krk island	35	N45°08'06"	E14°35'43"	5	AS
97	Kupica River spring, Mala Lešnica, NP Risnjak	270	N45°25'48"	E14°51'07"	5	BS
98	Mijića vrelo stream, Mijići	60	N44°09'37"	E15°52'38"	5	AS
99	Krupa River, Krupa	130	N44°11'34"	E15°54'34"	5	AS
100	Krupa River, Kudin bridge	90	N44°11'16"	E15°50'44"	5	AS
101	Pond, Zvjerinac	245	N43°56'45"	E16°12'56"	5	
102	Jaruga stream, Jelavića bridge, Zmijavci	260	N43°24'46"	E17°15'09"	5	AS
103	Otuča River, Deringaj, Kijani	615	N44°21'02"	E15°52'34"	5	AS
104	Vransko Lake, main channel, Biograd	0	N43°56'20"	E15°30'59"	5	AS
105	Vransko Lake, Biograd, Drage	5	N43°53'44"	E15°33'07"	5	AS
106	Krka River, Roški slap waterfall, NP Krka	75	N43°54'23"	E15°58'30"	5	AS
107	Visovac Lake, NP Krka	50	N43°51'38"	E15°58'55"	5	AS
108	Brljan Lake, NP Krka	205	N44°00'30"	E16°02'41"	5	AS
109*	Kosovčica River, upper reach, Vučenovići	230	N43°58'30"	E16°12'45"	5	AS
110*	Kosovčica River, lower reach, Biskupija	220	N44°00'26"	E16°12'52"	5	AS
111	Krka River, Knin	220	N44°01'56"	E16°11'26"	5	AS
112	Krka River, upstream of Kosovčica river mouth, Knin	220	N44°02'24"	E16°13'42"	5	AS
113	Krka River, downstream of Kosovčica river mouth, Knin	215	N44°01'41"	E16°12'48"	5	AS
114	Orašnica River, Knin	225	N44°01'56"	E16°12'04"	5	AS
115	Zrmanja River, Mokro polje, Prkos	200	N44°05'31"	E16°02'00"	5	AS
116	Zrmanja River, Vekići	130	N44°06'06"	E15°56'41"	5	AS
117	Zrmanja River, Palanka	270	N44°08'23"	E16°04'25"	5	AS
118	Zrmanja River, Muškovci, Berberi buk	20	N44°11'50"	E15°46'07"	5	AS
119	Zrmanja River, Kravlja Draga, bridge	240	N44°05'50"	E16°04'30"	5	AS
120	Zrmanja River, Žegar, bridge	60	N44°09'10"	E15°53'08"	5	AS
121	Zrmanja River, Draga	55	N44°09'50"	E15°50'43"	5	AS
122	Lopuško vrelo stream, Lake	220	N44°01'11"	E16°13'21"	5	AS
123	Krčić River, Kovačić	315	N44°02'19"	E16°16'42"	5	AS
124	Krčić River, Mlinica	380	N44°01'38"	E16°19'25"	5	AS
125	Šarena jezera lake, Biskupija	220	N44°01'36"	E16°13'22"	5	
126	Čikola River, near Rakići	100	N43°50'13"	E16°04'25"	5	AS
127	Čikola River, Otavice	270	N43°50'36"	E16°15'25"	5	AS
128	Vrba River, Vrba	425	N43°43'21"	E16°23'58"	5	AS
129	Vrba River, Čavoglave	290	N43°47'28"	E16°18'52"	5	AS
130	Butižnica River, Knin	220	N44°02'44"	E16°11'39"	5	AS
131	Brodic stream, Markovac, Biskupija	250	N43°57'03"	E16°15'00"	5	AS
132	Karakašica, Karakašica	320	N43°43'04"	E16°38'19"	5	AS
133	Boggy seepages, Bruvno, Gračac	690	N44°23'15"	E15°53'08"	5	
134	Ričina stream, Proložac	400	N43°29'20"	E17°09'11"	5	AS
135*	Cetina River, Spring Glavaš	385	N43°58'36"	E16°25'48"	5	AS
136	Grab River, Spring	330	N43°38'24"	E16°46'20"	5	AS
137*	Cetina River, Preočki most bridge	370	N43°57'59"	E16°25'53"	5	AS
138*	Cetina River, Crveni most bridge	365	N43°57'35"	E16°25'46"	5	AS
139*	Cetina River, Obrovac Sinjski	300	N43°43'58"	E16°41'11"	5	AS
140*	Cetina River, Trilj1	295	N43°36'54"	E16°43'42"	5	AS
141*	Cetina River, Čikotina lađa	250	N43°31'58"	E16°44'42"	5	AS
142*	Cetina River, Radmanove mlinice	15	N43°26'19"	E16°45'06"	5	AS
143*	Cetina River, Trilj2	295	N43°36'19"	E16°43'28"	5	AS
144	Cetina River, Peruča Reservoir	360	N43°47'45"	E16°35'32"	5	AS
145	Cetina River, Zadvarje	205	N43°26'02"	E16°53'18"	5	AS
146*	Ruda River, spring	295	N43°40'07"	E16°47'39"	5	AS
147*	Ruda River, upper reach	320	N43°40'06"	E16°47'28"	5	AS
148	Cetina River tributary stream, Vukovići, Paško polje	370	N43°58'06"	E16°25'07"	5	AS
149	Cetina River tributary stream, Kotluša, Paško polje	375	N43°56'54"	E16°24'06"	5	AS
150	Jadro River 1, Solin	10	N43°32'23"	E16°29'45"	5	AS
151	Matica River, Vrgorac	60	N43°12'21"	E17°23'46"	5	AS
152	Matica River, Umčani	40	N43°10'28"	E17°22'32"	5	AS
153	Stinjevac spring, Dusina	30	N43°10'29"	E17°25'02"	5	AS
154	Cetina River, Čitluk	300	N43°44'48"	E16°39'49"	5	AS
155	Vukovića vrilo spring, Bitelići, Hrvace	505	N43°49'12"	E16°37'28"	5	AS
156	Ljuta River, spring	90	N42°32'20"	E18°22'46"	5	AS
157	Ljuta River, upper reach, Donja Ljuta	60	N42°32'05"	E18°22'39"	5	AS
158	Vodovađa stream, Palje Brdo	110	N42°30'29"	E18°24'34"	5	AS
159	Konavočica River, near Karasovići	110	N42°30'19"	E18°24'37"	5	AS
160	Stream, near Zastolje	75	N42°31'17"	E18°23'31"	5	AS
161	Stream, near Brajkovići	90	N42°31'49"	E18°23'14"	5	AS
162	Vrljika River, Kamenmost	265	N43°25'52"	E17°11'42"	5	AS
163	Vrljika River, Kapuše	270	N43°26'33"	E17°10'32"	5	AS
164	Jarun Lake, Zagreb	110	N45°46'47"	E15°55'17"	11	BS
165○	Stream under the village Beram	290	N45°15'10"	E13°54'18"	5	AS
166○	Spring by the church, Stajnica, Porkulabi	500	N45°02'31"	E15°14'18"	5	AS
167○	Danube River, Ilok	75	N45°13'49"	E19°23'26"	11	BS
168○	Ljubica stream, Baške Oštarije, Linići, Velebit Mountain	910	N44°31'37"	E15°09'41"	5	AS
169○	Spring by the church, Slano	15	N42°47'01"	E17°53'26"	5	AS
170○	Spring by the sea, Dubrovnik, Mali Zaton	5	N42°42'06"	E18°02'40"	5	AS
171○	Tounjčica stream,Tounj	220	N45°14'56"	E15°20'04"	5	BS

* Sampling sites used in calculating Shannon-Weaver and Simpson indices and in Cluster analysis.

○ Samples stored in Slovene Natural History Museum. The remaining samples are stored at the University of Zagreb, Faculty of Science, Department of Biology, Division of Zoology, Zagreb.

Mayflies were sampled in every season at 34 sites, while at the remainder of sites, sampling was usually performed only once between April and September. Specimens were stored in 80% ethanol and identified in the lab using a stereomicroscope and microscope. A reference collection was made by preparing permanent slide mounts of identified species. Larvae were treated with 10% KOH and 99% acetic acid to remove all muscle parts. Mouth parts, legs, gills, thorax, abdomen, paraproct plate in Baetidae and cerci, necessary for the species identification, were fixed in Euparal and examined under a microscope. Adult specimens were mostly identified by the imaginal male genitalia. The collected material (larvae and adult specimens) was identified using Müller-Liebenau (1969), Elliott and Humpesch (1983), Malzacher (1984), Elliott et al. (1988), Studemann et al. (1992), Haybach (1999), Bauernfeind and Humpesch (2001), combined with numerous publications with species descriptions (e.g. Tomka and Rasch 1993).

### Data analysis

All recorded specimens were included in the Croatian mayfly species list. Data for the sites with the same sampling effort were statistically analysed using the PRIMER 6 software package (Clarke and Warwick 2001). As such, only 34 sampling sites were compared out of the total 171 (Table 1). These sites were sampled in all seasons, at the available microhabitats and they represent habitats in each ecoregion and each sea basin. Species diversity, evenness, and similarity between sites with respect to the mayfly composition and abundance were determined by the Shannon-Weaver and Simpson indices. For estimation of similarity and differences in the mayfly community composition, cluster analysis was used. Similarity among sites was determined using the Bray-Curtis similarity index. SIMPER (Similarity Percentage) was used to assess which taxa are primarily responsible for the similarities between the sites of the same habitat type. The Croatian mayfly species richness was compared with the surrounding countries (Bosnia & Herzegovina, Hungary, Slovenia, Italy) by compiling species list for these countries taken from Bauernfeind and Soldán (2012) and the Sørensen Index of Similarity was calculated.

## Results

### Species richness

In total, 79 mayfly taxa (Table 2) were recorded for Croatia. Of the 171 sites (55 in ER11, 116 in ER5) investigated during this study (Table 1), 66 taxa were sampled, of which 29 were recorded for the first time (Table 2). The presence of 13 (16%) previously recorded species could not be confirmed (Table 2). The most diverse genera were *Baetis* Leach, 1815 and *Ecdyonurus* Eaton, 1868 both with 11 species. *Baetis rhodani* (Pictet, 1843) and *Serratella ignita* (Poda, 1761) were the most widely distributed species, present in 83 and 76 sampling sites, respectively. Fourteen species were recorded at only one sampling site: *Cloeon simile* Eaton, 1870, *Procloeon nana* (Bogoescu, 1951), *Caenis pusilla* Navàs, 1913, Ephemera cf. parnassiana Demoulin, 1958, *Leptophlebia vespertina* (Linnaeus, 1758), *Ecdyonurus vitoshensis* Jacob & Braasch, 1984, *Ecdyonurus zelleri* (Eaton, 1885), *Electrogena mazedonica* (Ikonomov, 1954), *Heptagenia coerulans* Rostock, 1878, *Heptagenia flava* Rostock, 1878, *Heptagenia longicauda* (Stephens, 1835), *Rhithrogena iridina* (Kolenati, 1839), Rhithrogena gr. diaphana and *Rhithrogena semicolorata* (Curtis, 1834).

**Table 2.** Croatian mayfly fauna.

Mayfly taxa	Ecoregion	Habitat type	Basin
Ametropodidae			
■*Ametropus fragilis* Albarda, 1878	11	3	BS
Ameletidae			
▲*Ameletus inopinatus* Eaton, 1887	-	-	-
▲*Metreletus balcanicus* (Ulmer, 1920)	-	-	-
Siphlonuridae			
▲*Siphlonurus armatus* (Eaton, 1870)	-	-	-
*Siphlonurus croaticus* Ulmer, 1920	11	2,3,4	AS
*Siphlonurus lacustris* (Eaton, 1870)	5, 11	2,3	BS, AS
Baetidae			
*Alainites muticus* (Linnaeus, 1758)	5	2,3,4	BS, AS
*Baetis alpinus* (Pictet, 1843)	5, 11	1,2,3	BS
●*Baetis buceratus* Eaton, 1870	11	3	BS
*Baetis fuscatus* (Linnaeus, 1761)	5, 11	3	BS
●*Baetis liebenauae* Keffermüller, 1974	5, 11	1,2,3	BS, AS
*Baetis lutheri* Müller-Liebenau, 1967	5, 11	1,3	BS, AS
●*Baetis melanonyx* (Pictet, 1843)	5	1,2,3	AS
●Baetis cf. nubecularis (Eaton, 1898)	5	1,2,3,4	BS
*Baetis rhodani* (Pictet, 1843)	5, 11	1,2,3,4	BS, AS
*Baetis scambus* Eaton, 1870	11	3	BS
●*Baetis tricolor* Tshernova, 1928	11	3	BS
●*Baetis vernus* Curtis, 1834	5, 11	3	BS, AS
●*Baetopus tenellus* (Albarda, 1878)	5, 11	2,3	BS
●*Nigrobaetis niger* (Linnaeus, 1761)	5, 11	2,3	BS, AS
*Centroptilum luteolum* (Müller, 1776)	5, 11	2,3,4,5	BS, AS
*Cloeon dipterum* (Linnaeus, 1761)	5, 11	2,3,5	BS, AS
*Cloeon simile* Eaton, 1870	5	5	AS
*Procloeon bifidum* (Bengtsson, 1912)	5, 11	2,3	BS, AS
●*Procloeon nana* (Bogoescu, 1951)	5	2	AS
*Procloeon pennulatum* (Eaton, 1870)	5, 11	3,4	BS, AS
Caenidae			
▼*Brachycercus harrisellus* Curtis, 1834	11	3	BS
●*Caenis beskidensis* Sowa, 1973	5	3	AS
*Caenis horaria* (Linnaeus, 1758)	5, 11	3,4,5	BS, AS
*Caenis macrura* Stephens, 1835	5, 11	3	BS, AS
●*Caenis pusilla* Navàs, 1913	5	3	BS
●*Caenis rivulorum* Eaton, 1884	11	3	BS
●*Caenis robusta* Eaton, 1884	11	2,3,5	BS
Ephemerellidae			
●*Ephemerella mucronata* (Bengtsson, 1909)	5, 11	2,3	BS, AS
*Serratella ignita* (Poda, 1761)	5, 11	1,2,3,4	BS, AS
*Torleya major* (Klapalek, 1905)	5, 11	2,3,4	BS, AS
Ephemeridae			
*Ephemera danica* Müller, 1764	5, 11	2,3,4,5	BS, AS
▲*Ephemera glaucops* Pictet, 1843	-	-	-
*Ephemera lineata* Eaton, 1870	5	2,3,5	AS
●Ephemera cf. parnassiana Demoulin, 1958	5	2	AS
*Ephemera vulgata* Linnaeus, 1758	5, 11	2,3,5	BS, AS
●*Ephemera zettana* Kimmins, 1937	5	2,3	AS
Palingeniidae			
▲*Palingenia longicauda* (Olivier, 1791)	-	-	-
Polymitarcyidae			
▲*Ephoron virgo* (Olivier, 1791)	-	-	-
Leptophlebiidae			
▲*Choroterpes picteti* (Eaton, 1871)	-	-	-
*Habroleptoides confusa* Sartori and Jacob, 1986	5, 11	2,3	BS, AS
*Habrophlebia fusca* (Curtis, 1834)	5, 11	1,2,3	BS, AS
*Habrophlebia lauta* Eaton, 1884	5, 11	2,3,5	BS, AS
●*Leptophlebia vespertina* (Linnaeus, 1758)	5	2,5	BS, AS
*Paraleptophlebia submarginata* (Stephens, 1835)	5, 11	2,3,4	BS, AS
●*Paraleptophlebia werneri* Ulmer, 1920	5	2,5	BS
Oligoneuriidae			
*Oligoneuriella rhenana* (Imhoff, 1852)	11	3	BS
Potamanthidae			
*Potamanthus luteus* (Linnaeus, 1767)	11	3	BS
Heptageniidae			
▲*Ecdyonurus aurantiacus* (Burmeister, 1839)	-	-	-
*Ecdyonurus dispar* (Curtis, 1834)	5	2,3	BS, AS
*Ecdyonurus insignis* (Eaton, 1870)	5, 11	3	BS, AS
●*Ecdyonurus macani* Thomas & Sowa, 1970	5, 11	3	BS, AS
▲*Ecdyonurus siveci* Hefti, Tomka & Zurwerra, 1986	-	-	-
●*Ecdyonurus starmachi* Sowa, 1971	5, 11	2,3	BS, AS
●*Ecdyonurus submontanus* Landa, 1969	5	3	BS
*Ecdyonurus torrentis* Kimmins, 1942	5	2,3	BS, AS
*Ecdyonurus venosus* (Fabricius, 1775)	5	2,3	AS
*Ecdyonurus vitoshensis* Jacob & Braasch, 1984	11	2	BS
●*Ecdyonurus zelleri* (Eaton, 1885)	11	2	BS
●*Electrogena affinis* (Eaton, 1883)	5	2,3	AS
*Electrogena lateralis* (Curtis, 1834)	5, 11	2,3,4	BS, AS
●*Electrogena mazedonica* (Ikonomov, 1954)	5	3	AS
●*Electrogena ujhelyii* (Sowa, 1981)	5, 11	1,2	BS, AS
*Epeorus assimilis* Eaton, 1885	5, 11	1,2,3	BS, AS
*Heptagenia coerulans* Rostock, 1878	11	3	BS
*Heptagenia flava* Rostock, 1878	11	3	BS
●*Heptagenia longicauda* (Stephens, 1835)	5	3	BS
*Heptagenia sulphurea* (Müller, 1776)	11	3	BS
▲*Kageronia fuscogrisea* (Retzius, 1783)	-	-	-
●*Rhithrogena braaschi* Jacob, 1974	5	1,2,3	BS, AS
●Rhithrogena gr. diaphana	11	3	BS
▲*Rhithrogena germanica* Eaton, 1885	-	-	-
●*Rhithrogena iridina* (Kolenati, 1839)	11	2	BS
*Rhithrogena semicolorata* (Curtis, 1834)	11	2	BS

▲ Only literature data: Bauernfeind and Soldán (2012) - presence in Croatia noted without referent to exact localities.

▼ Only literature data: Kovács and Murányi (2013).

■ Only literature data: Ćuk et al. (2015).

● New records for the Croatian mayfly fauna.

Ecoregion: 5 = Dinaric western Balkan, 11 = Pannonian lowland.

Habitat type: 1 = spring, 2 = stream, 3 = river, 4 = tufa barrier, 5 = lake, - = unknown/missing data.

Basin: BS = Black Sea Basin ; AS = Adriatic Sea Basin .

Approximately half of the species (30) were present in both ecoregions. A total of 50 species was recorded as present only in the Dinaric western Balkan ecoregion (ER5) and 48 only in the Pannonian lowland ecoregion (ER11) (Table 2). Nearly half the species (32) were recorded in both the Black and Adriatic Sea Basins, while 25 species were recorded only for Black Sea basin and 11 species only for Adriatic Sea basin (Table 2).

The Sørensen Index of Similarity indicated the Croatian mayfly fauna had the greatest similarity with the Hungarian assemblage (Table 3).

**Table 3.** Sørensen Index of Similarity between mayfly assemblages for surrounding countries in relation to Croatia. CRO = Croatia, B&H = Bosnia and Herzegovina, I = Italy, SLO = Slovenia, HUN = Hungary.

	CRO	B&H	I	SLO
CRO				
B&H	64.62			
I	55.44	51.89		
SLO	61.64	56.67	51.72	
HUN	74.85	60.69	54.27	52.17

### Mayflies (Insecta, Ephemeroptera) of Croatia

For the distribution data, the following format was used: “Literature data” were mainly taken from Bauernfeind and Soldán (2012), which listed the presence of each species in Croatia but without reference to their exact localities. Two and one species and localities where they were recorded were mentioned in Kovács and Murányi (2013) and Ćuk et al., respectively. “Literature data with new records” corresponds to data obtained as a part of this study but were already published. “New records” are data obtained in this study but were not yet published. For every species, the site ID is listed. All sampling sites and their ID numbers are listed in Table 1.

● New records for the Croatian mayfly fauna

■ Only adults recorded

**I. Ametropodidae Bengtsson, 1913**

1. *Ametropus fragilis* Albarda, 1878

**Literature data**: Drava River, Donji Miholjac (Ćuk et al. 2015)

**II. Ameletidae McCafferty, 1991**

2. *Ameletus inopinatus* Eaton, 1887

**Literature data**: Bauernfeind and Soldán (2012)

3. *Metreletus balcanicus* (Ulmer, 1920)

**Literature data**: Bauernfeind and Soldán (2012)

**III. Baetidae Leach, 1815**

4. *Alainites muticus* (Linnaeus, 1758)

**Literature data**: Bauernfeind and Soldán (2012)

**Literature data with new records**: 79, 80■, 82, 84, 85, 86 (Vilenica et al. 2014)

**New records**: 68, 70, 115, 150, 158, 160, 161, 162, 163,165, 168

5. *Baetis alpinus* (Pictet, 1843)

**Literature data**: Bauernfeind and Soldán (2012)

**New records**: 13, 15, 57, 63

6. *Baetis buceratus* Eaton, 1870 ●

**New records**: 2, 36

7. *Baetis fuscatus* (Linnaeus, 1761)

**Literature data**: Bauernfeind and Soldán (2012)

**New records**: 5, 7, 8, 10, 18, 19, 26, 29, 31, 32, 35, 36, 40, 56, 60, 61, 62

8. *Baetis liebenauae* Keffermüller, 1974 ●

**New records**: 1, 2, 9, 10, 35, 36, 37, 62, 98, 109, 110, 111, 112, 113, 122, 128, 131, 134, 139, 140, 141, 143, 151, 152, 153, 162, 171

9. *Baetis lutheri* Müller-Liebenau, 1967

**Literature data**: Bauernfeind and Soldán (2012)

**New records**: 7, 18, 19, 35, 61, 62, 103, 116, 141, 142, 146, 147, 150, 157

10. *Baetis melanonyx* (Pictet, 1843) ●

**New records**: 115, 117, 120, 146, 147, 156, 157, 158, 159, 160, 161, 162, 163

11. Baetis cf. nubecularis Eaton, 1898 ●

**Literature data with new records**: 79, 80, 81, 82, 83, 84, 85, 86, 87 (Vilenica et al. 2014)

12. *Baetis rhodani* (Pictet, 1843)

**Literature data**: Bauernfeind and Soldán (2012)

**Literature data with new records**: 79, 80, 81, 82, 83, 84, 85, 87, 88 (Vilenica et al. 2014)

**New records**: 9, 10, 13, 15, 16, 23, 24, 26, 28, 29, 30, 31, 32, 34, 35, 48, 50, 51, 53, 59, 61, 62, 63, 64, 65, 66, 68, 70, 77, 78, 98, 99, 100, 103, 109, 110, 112, 113, 114, 115, 116, 117, 118, 120, 122, 123, 124, 128, 131, 132, 134, 135, 137, 138, 139, 140, 141, 142, 146, 147, 148, 149, 153, 157, 158, 159, 160, 161, 162, 163, 166, 169, 170, 171

13. *Baetis scambus* Eaton, 1870

**Literature data**: Bauernfeind and Soldán (2012)

**New records**: 7, 26

14. *Baetis tricolor* Tshernova, 1928 ●

**New records**: 20, 43, 44

15. *Baetis vernus* Curtis, 1834 ●

**New records**: 7, 9, 10, 36, 38, 53, 54, 76

16. *Baetopus tenellus* (Albarda, 1878) ●

**New records**: 19, 64, 94

17. *Nigrobaetis niger* (Linnaeus, 1761) ●

**Literature data with new records**: 138

**New records**: 15, 36, 38, 93, 103, 109, 110, 128, 131

18. *Centroptilum luteolum* (Müller, 1776)

**Literature data**: Bauernfeind and Soldán (2012)

**Literature data with new records**: 84, 85, 86, 87, 88, 89, 90, 91, 92 (Vilenica et al. 2014)

**New records**: 1, 12, 23, 27, 28, 31, 32, 35, 61, 62, 69, 74, 77, 78, 103, 107, 109, 110, 121, 127, 128, 141, 142, 143, 144, 159

19. *Cloeon dipterum* (Linnaeus, 1761)

**Literature data**: Bauernfeind and Soldán (2012)

**New records**: 1, 5, 20, 24, 35, 37, 39, 41, 43, 44, 45, 46, 47, 60, 67, 78, 101, 103, 104, 105, 121, 125, 127, 128, 129, 152

20. *Cloeon simile* Eaton, 1870

**Literature data**: Bauernfeind and Soldán (2012)

**New records**: 125

21. *Procloeon bifidum* (Bengtsson, 1912)

**Literature data**: Bauernfeind and Soldán (2012)

**New records**: 6, 19, 20, 28, 29, 31, 32, 40, 41, 42, 44, 47, 62, 68, 69, 71, 115, 121, 141

22. *Procloeon nana* (Bogoescu, 1951) ●

**New records**: 68

23. *Procloeon pennulatum* (Eaton, 1870)

**Literature data**: Bauernfeind and Soldán (2012)

**Literature data with new records**: 84, 85, 86 (Vilenica et al. 2014)

**New records**: 26, 27, 61, 127, 129

**IV. Caenidae Newman, 1853**

24. *Brachycercus harrisellus* Curtis, 1834

**Literature data**: Vojlovica River at the bridge of road No. 2, Vojlovica (Kovács and Murányi 2013)

25. *Caenis beskidensis* Sowa, 1973 ●

**New records**: 139, 140, 141, 143, 142

26. *Caenis horaria* (Linnaeus, 1758)

**Literature data**: Bauernfeind and Soldán (2012)

**Literature data with new records**: 86, 87, 89, 90, 91, 92 (Vilenica et al. 2014)

**New records**: 39, 73, 78, 101, 106, 107

27. *Caenis macrura* Stephens, 1835

**Literature data**: Bauernfeind and Soldán (2012)

**New records**: 8, 9, 10, 18, 26, 27, 28, 31, 32, 35, 40, 41, 54, 61, 68, 71, 115, 140, 141, 142, 143

28. *Caenis pusilla* Navàs, 1913 ●

**New records**: 62

29. *Caenis rivulorum* Eaton, 1884 ●

**New records**: 40, 41

30. *Caenis robusta* Eaton, 1884 ●

**New records**: 1, 24, 39, 47

**V. Ephemerellidae Klapálek, 1909**

31. *Ephemerella mucronata* (Bengtsson, 1909) ●

**New records**: 14, 134, 139, 163

32. *Serratella ignita* (Poda, 1761)

**Literature data**: Bauernfeind and Soldán (2012)

**Literature data with new records**: 83, 84, 85, 86, 88 (Vilenica et al. 2014))

**New records**: 1, 7, 8, 9, 10, 12, 17, 26, 27, 28, 29, 30, 31, 32, 34, 35, 36, 37, 48, 49, 53, 46, 58, 59, 60, 61, 62, 64, 65, 66, 68, 69, 73, 76, 98, 99, 100, 103, 108, 109, 110, 113, 114, 115, 116, 117, 118, 119, 121, 122, 129, 134, 137, 138, 139, 140, 141, 142, 143, 144, 146, 147, 148, 150, 153, 157, 158, 159, 162, 163, 171

33. *Torleya major* (Klapalek, 1905)

**Literature data**: Bauernfeind and Soldán (2012)

**Literature data with new records**: 84, 86 (Vilenica et al. 2014)

**New records**: 53, 66, 117, 118, 139, 141

**VI. Ephemeridae Latreille, 1810**

34. *Ephemera danica* Müller, 1764

**Literature data**: Bauernfeind and Soldán (2012)

**Literature data with new records**: 82, 83, 84, 85, 86, 87, 88, 89, 90, 91, 92 (Vilenica et al. 2014)

**New records**: 8, 14, 17, 23, 27, 28, 30, 33, 48, 49, 53, 59, 60, 61, 63, 64, 66, 68, 95, 100, 115,141, 142

35. *Ephemera glaucops* Pictet, 1843

**Literature data**: Bauernfeind and Soldán (2012)

36. *Ephemera lineata* Eaton, 1870

**Literature data**: Bauernfeind and Soldán (2012)

**New records**: 106, 107, 108, 109, 110, 118, 119, 122, 137, 138, 139, 140, 141, 142, 143, 147

37. Ephemera cf. parnassiana Demoulin, 1958 ●

**New records**: 98

38. *Ephemera vulgata* Linnaeus, 1758

**Literature data**: Bauernfeind and Soldán (2012)

**New records**: 11, 54, 55, 59, 100, 125, 128, 154, 164

39. *Ephemera zettana* Kimmins, 1937 ● ■

**New records**: 102, 118, 134, 136, 138, 141, 142, 154, 155

**VII. Heptageniidae Needham, 1901**

40. *Ecdyonurus aurantiacus* (Burmeister, 1839)

**Literature data**: Bauernfeind and Soldán (2012)

41. *Ecdyonurus dispar* (Curtis, 1834)

**Literature data**: Bauernfeind and Soldán (2012)

**New records**: 61, 63, 66, 68, 69

42. *Ecdyonurus insignis* (Eaton, 1870)

**Literature data**: Cetina River, between Podgrade and Slime (Kovács and Murányi 2013)

**New records**: 26, 27, 32, 116, 141, 145

43. *Ecdyonurus macani* Thomas & Sowa, 1970 ●

**New records**: 7, 26, 27, 137, 138, 139, 141, 147

44. *Ecdyonurus siveci* Hefti, Tomka & Zurwerra, 1986

**Literature data**: Bauernfeind and Soldán (2012)

45. *Ecdyonurus starmachi* Sowa, 1971 ●

**New records**: 13, 14, 26, 53, 103, 120

46. *Ecdyonurus submontanus* Landa, 1969 ●

**Literature data with new records**: 82, 83 (Vilenica et al. 2014)

47. *Ecdyonurus torrentis* Kimmins, 1942

**Literature data**: Bauernfeind and Soldán (2012)

**New records**: 95, 99, 118, 119, 120

48. *Ecdyonurus venosus* (Fabricius, 1775)

**Literature data**: Bauernfeind and Soldán (2012)

**New records**: 97■, 99, 100, 109, 110, 112, 118, 119, 120, 137, 138, 139, 141, 148, 150, 162

49. *Ecdyonurus vitoshensis* Jacob & Braasch, 1984

**Literature data**: Bauernfeind and Soldán (2012)

**New records**: 12

50. *Ecdyonurus zelleri* (Eaton, 1885) ●

**New records**: 53

51. *Electrogena affinis* (Eaton, 1883) ●

**New records**: 68, 69, 70

52. *Electrogena lateralis* (Curtis, 1834)

**Literature data**: Bauernfeind and Soldán (2012)

**Literature data with new records**: 86 (Vilenica et al. 2014)

**New records**: 12, 27, 61, 96, 165

53. *Electrogena mazedonica* (Ikonomov, 1954) ●

**New records**: 128

54. *Electrogena ujhelyii* (Sowa, 1981) ●

**New records**: 11, 13, 16, 24, 50, 93

55. *Epeorus assimilis* Eaton, 1885

**Literature data**: Bauernfeind and Soldán (2012)

**New records**: 4, 13, 94, 97■, 98, 99, 115, 116, 117, 120, 135■, 137, 138, 141, 142, 146, 147, 156

56. *Heptagenia coerulans* Rostock, 1878

**Literature data**: Bauernfeind and Soldán (2012)

**New records**: 18

57. *Heptagenia flava* Rostock, 1878

**Literature data**: Bauernfeind and Soldán (2012)

**New records**: 167

58. *Heptagenia longicauda* (Stephens, 1835) ●

**New records**: 63

59. *Heptagenia sulphurea* (Müller, 1776)

**Literature data**: Bauernfeind and Soldán (2012)

**New records**: 7, 8, 18, 21, 40, 42

60. *Kageronia fuscogrisea* (Retzius, 1783)

**Literature data**: Bauernfeind and Soldán (2012)

61. *Rhithrogena braaschi* Jacob, 1974 ●

**Literature data with new records**: 79, 80, 81, 82, 83, 85 (Vilenica et al. 2014)

**New records**: 57, 58, 109, 110, 112, 117, 120, 122, 124, 135, 137, 138, 139, 141, 142, 143, 146, 147, 162, 163

62. Rhithrogena gr. diaphana ●

**New records**: 32

63. *Rhithrogena germanica* Eaton, 1885

**Literature data**: Bauernfeind and Soldán (2012)

64. *Rhithrogena iridina* (Kolenati, 1839) ●

**New records**: 27

65. *Rhithrogena semicolorata* (Curtis, 1834)

**Literature data**: Bauernfeind and Soldán (2012)

**New records**: 53

**VIII. Leptophlebiidae Banks, 1900**

66. *Choroterpes picteti* (Eaton, 1871)

**Literature data**: Bauernfeind and Soldán (2012)

67. *Habroleptoides confusa* Sartori and Jacob, 1986

**Literature data**: Bauernfeind and Soldán (2012)

**New records**: 22, 120, 158

68. *Habrophlebia fusca* (Curtis, 1834)

**Literature data**: Bauernfeind and Soldán (2012)

**New records**: 27, 28, 30, 35, 38, 48, 59, 69, 70, 131, 168, 169

69. *Habrophlebia lauta* Eaton, 1884

**Literature data**: Bauernfeind and Soldán (2012)

**Literature data with new records**: 82, 83, 85, 90 (Vilenica et al. 2014)

**New records**: 25, 26, 27, 29, 48, 49, 61, 65, 66, 68, 70, 109, 110

70. *Leptophlebia vespertina* (Linnaeus, 1758) ●

**Literature data with new records**: 90, 91 (Vilenica et al. 2014)

**New records**: 134

71. *Paraleptophlebia submarginata* (Stephens, 1835)

**Literature data**: Bauernfeind and Soldán (2012)

**Literature data with new records**: 79, 83, 84, 85, 86, 87, 88 (Vilenica et al. 2014)

**New records**: 8, 14, 26, 53, 60, 61, 74, 77, 98, 109, 110, 118, 119, 120, 128, 134, 137, 138, 139, 141, 142, 162

72. *Paraleptophlebia werneri* Ulmer, 1920 ●

**Literature data with new records**: 85, 90 (Vilenica et al. 2014)

**IX. Oligoneuriidae Ulmer, 1914**

73. *Oligoneuriella rhenana* (Imhoff, 1852)

**Literature data**: Bauernfeind and Soldán (2012)

**New records**: 26, 27, 32

**X. Palingeniidae Albarda, 1888**

74. *Palingenia longicauda* (Olivier, 1791)

**Literature data**: Bauernfeind and Soldán (2012)

**XI. Polymitarcyidae Banks, 1900**

75. *Ephoron virgo* (Olivier, 1791)

**Literature data**: Bauernfeind and Soldán (2012)

**XII. Potamanthidae Albarda, 1888**

76. *Potamanthus luteus* (Linnaeus, 1767)

**Literature data**: Bauernfeind and Soldán (2012)

**New records**: 7, 8, 9, 10, 18, 35, 36, 37, 40

**XIII. Siphlonuridae Ulmer, 1920 (1888)**

77. *Siphlonurus armatus* (Eaton, 1870)

**Literature data**: Bauernfeind and Soldán (2012)

78. *Siphlonurus croaticus* Ulmer, 1920

**Literature data**: Bauernfeind and Soldán (2012)

**Literature data with new records**: 82, 83, 85, 87 (Vilenica et al. 2014)

**New records**: 55, 66, 111, 123, 128, 130, 135■, 137

79. *Siphlonurus lacustris* (Eaton, 1870)

**Literature data**: Bauernfeind and Soldán (2012)

**New records**: 26, 27, 30, 73, 76

## Community composition

**Figure 2.**
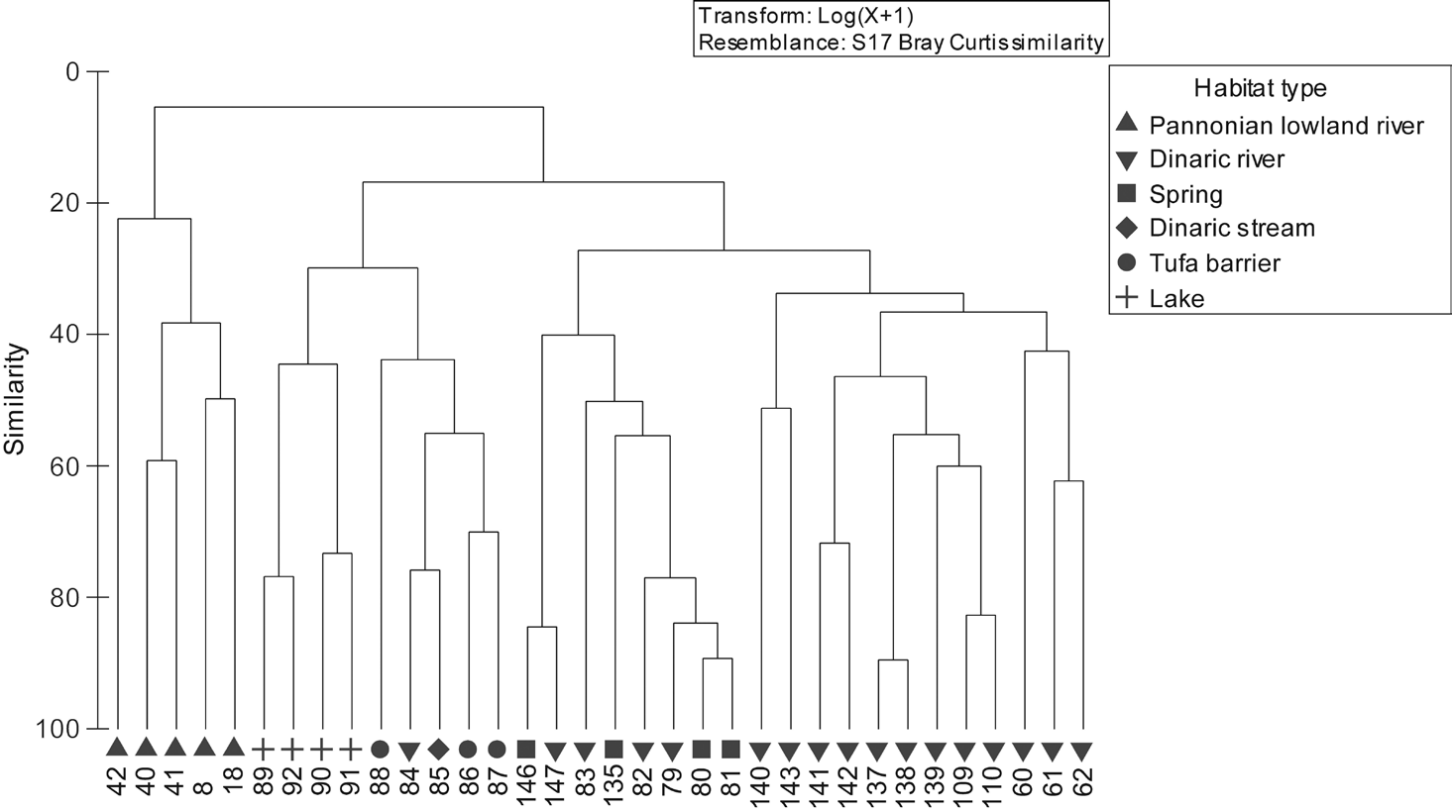
Cluster analysis of mayfly community composition, based on Bray-Curtis Similarity (See Table 1 for codes).

The majority of the Croatian mayfly species were found to be associated with rivers and streams (Table 2). Among these, larvae of ten species also occurred within the spring areas (Table 2). Eleven species recorded in lakes and/or ponds were also found to inhabit flowing-water habitats. Cluster analysis (Fig. 2) showed that based on the mayfly assemblage, sampling sites were mainly structured first by ecoregion and then by habitat type. Species richness at the sampling sites and diversity indices are presented in Table 4. Species richness ranged from 2 and 18 species, Shannon-Weaver index between 0.21 and 1.96 and Simpson index between 0.11 and 0.82. All sampling sites with the highest species richness and diversity indices were situated in the Dinaric western Balkan ecoregion (ER5).

**Table 4.** Species richness (S), Shannon-Weaver (H’) and Simpson (1-λ) indices of diversity, calculated for 34 sites. Sites with the highest H’ and 1-λ are in bold.

Sampling site	S	H’	1-λ
8	7	1.38	0.65
18	6	1.05	0.54
40	6	1.19	0.62
41	5	1.09	0.55
42	2	0.56	0.4
60	5	0.31	0.12
61	11	0.76	0.31
62	8	0.85	0.44
79	5	0.95	0.56
80	4	1.01	0.61
81	3	0.98	0.59
82	7	0.77	0.39
83	10	**1.70**	**0.75**
84	9	1.43	0.69
85	12	**1.67**	**0.75**
86	10	1.51	0.71
87	7	1.41	0.67
88	5	1.06	0.59
89	3	0.86	0.56
90	6	0.52	0.24
91	4	0.86	0.43
92	3	1.06	0.66
109	10	**1.77**	**0.75**
110	9	1.42	0.69
135	2	0.21	0.11
137	9	1.30	0.66
138	9	1.26	0.65
139	11	1.35	0.61
140	6	1.31	0.65
141	18	**1.96**	**0.81**
142	11	**1.83**	**0.82**
143	7	1.09	0.52
146	4	1.09	0.59
147	8	1.08	0.56

The SIMPER analysis between sites within the same habitat type showed an average similarity ranging from 35.1% for the Pannonian lowland rivers to 57.3% for the springs (Table 5).

**Table 5.** SIMPER analysis for similarities in mayfly community composition in different habitat types (Pannonian lowland river, Dinaric river, Spring, Tufa barrier, Lake). Average similarity reflects the percentage between samples within one habitat type.

Habitat type	Average similarity	Taxa	Av.Abund	Av.Sim	Sim/SD	Contrib%	Cum.%
Pannonian lowland river	35.10	*Caenis macrura*	3.56	10.09	1.12	28.76	28.76
		*Heptagenia sulphurea*	2.64	9.02	0.95	25.69	54.45
		*Potamanthus luteus*	2.72	7.64	0.98	21.77	76.22
		*Procloeon bifidum*	0.98	3.74	0.58	10.66	86.88
		*Caenis rivulorum*	1.46	2.32	0.32	6.61	93.49
Dinaric river	37.92	*Serratella ignita*	4.64	11.97	1.47	31.57	31.57
		*Baetis rhodani*	4.46	10.05	1.49	26.49	58.06
		*Rhithrogena braaschi*	3.16	5.18	0.73	13.67	71.73
		*Paraleptophlebia submarginata*	1.85	2.41	0.69	6.35	78.08
		*Ephemera lineata*	1.62	1.68	0.59	4.43	82.51
		*Baetis liebenauae*	1.1	0.99	0.4	2.6	85.11
		*Baetis lutheri*	1.45	0.89	0.27	2.36	87.47
		*Centroptilum luteolum*	1.04	0.64	0.45	1.7	89.16
		*Ephemera danica*	0.94	0.6	0.31	1.58	90.74
Spring	57.32	*Rhithrogena braaschi*	5.21	33.1	3.43	57.75	57.75
		*Baetis rhodani*	4.44	20.02	3.11	34.93	92.67
Tufa barrier	53.92	*Ephemera danica*	4.66	18.86	12.75	34.98	34.98
		*Paraleptophlebia submarginata*	2.99	11.45	9.54	21.24	56.21
		*Centroptilum luteolum*	2.85	8.47	2.05	15.7	71.92
		*Baetis rhodani*	2.31	6.07	0.58	11.26	83.18
		Baetis cf. nubecularis	2.94	5.71	0.58	10.59	93.77
Lake	54.64	*Caenis horaria*	4.44	21.65	2.46	39.63	39.63
		*Ephemera danica*	2.42	16.91	2.67	30.96	70.59
		*Centroptilum luteolum*	3.08	13.41	1.9	24.55	95.14

Av. abund. = average abundance , av. sim. = average similarity , Sim/SD = standard deviation of similarity , Contrib% = contribution to similarity , cum.% = cumulative percentage of similarity .

## Discussion

Due to the paucity of systematic studies, mayfly fauna and their habitat preferences in Croatia were very poorly known, with records of only 50 species (Bauernfeind and Soldán 2012, Kovács and Murányi 2013, Ćuk et al. 2015). As expected, this study showed a higher diversity: 66 taxa were recorded, of which 29 for the first time in Croatia (Table 2). Combined with the literature, the species list consists of 79 taxa. Croatia is a relatively small Balkan country divided into two Ecoregions: Dinaric western Balkan (ER5) and Pannonian lowland (ER11) (Illies 1978) due to its position on the crossroads of Central and Mediterranean Europe, which is why its mayfly fauna shows transitive characteristics.

As a result, species with wide (e.g. *Baetis rhodani*, *Cloeon dipterum*, *Caenis horaria*, *Serratella ignita*), patchy (e.g. *Procloeon nana*, *Leptophlebia vespertina*, *Caenis beskidensis*) central European (e.g. Baetis cf. nubecularis, *Ecdyonurus zelleri*, *Electrogena ujhelyii*) as well as southern (e.g. *Ephemera zettana*) and Balkan (e. g. *Electrogena mazedonica*, *Rhithrogena braaschi*, Ephemera cf. parnassiana) distribution were recorded in Croatia. Additionally, 15 taxa were found that were not previously recorded in the Dinaric western Balkan ecoregion: Baetis cf. nubecularis, *Procloeon nana*, *Caenis beskidensis*, Ephemera cf. parnassiana, *Ecdyonurus macani*, *Ecdyonurus submontanus*, *Ecdyonurus torrentis*, *Electrogena affinis*, *Electrogena mazedonica*, *Electrogena ujhelyii*, *Heptagenia longicauda*, *Rhithrogena braaschi*, *Habroleptiodes confusa*, *Leptophlebia vespertina* and *Paraleptophlebia werneri* (Buffagni et al. 2007, 2009, Bauernfeind and Soldán 2012).

The new records include several morphologically interesting taxa: *Rhithrogena* from the *diaphana* group, Baetis cf. nubecularis and Ephemera cf. parnassiana. The *Rhithrogena* species from the *diaphana* group is morphologically similar to *Rhithrogena savoiensis* Alba-Tercedor & Sowa, 1987. However, DNA analysis based on mitochondrial COI gene shows it to be more closely related to *Rhithrogena beskidensis* Alba-Tercedor & Sowa, 1987 (Vuataz unpubl. results). Thus, reliable identification cannot be distinguished at this time. Comparison with other Balkan *Rhithrogena diaphana* group species and further detailed studied are required. A similar case is recorded for the *Baetis alpinus* group (sensu Müller-Liebenau, 1969), which presents the morphological characteristics that are intermediate between *Baetis alpinus* and *Baetis nubecularis*. Interestingly, the species is only recorded in high numbers (Vilenica et al. 2014) in the mountain Dinaric karst streams and tufa barriers in the area of Plitvice Lakes National Park (Table 1, Fig. 1). One male imago of the genus *Ephemera* Linnaeus, 1758, was caught in the Lopoško vrelo stream in southern Croatia. Its morphological features correspond to *Ephemera parnassiana*, a species that has currently only been recorded from Greece; however due to the small sample size, additional specimens are necessary for accurate identification of the species.

As most sites were in running waters and often with a stony substrate, the most diverse genera were *Baetis* and *Ecdyonurus*, which are known to be very common in running waters of the Northern Hemisphere (Bauernfeind and Soldán 2012). The most widely distributed species were two eurytopic and eurythermic species: *Baetis rhodani* and *Serratella ignita*. Further study is required at new sampling sites to determine the distribution of eleven species recorded only at only a single sampling site (*Cloeon simile*, *Procloeon nana*, *Caenis pusilla*, Ephemera cf. parnassiana, *Leptophlebia vespertina*, *Ecdyonurus vitoshensis*, *Ecdyonurus zelleri*, *Electrogena mazedonica*, *Heptagenia coerulans*, *Heptagenia flava*, *Heptagenia longicauda*, *Rhithrogena iridina*, Rhithrogena gr. diaphana and *Rhithrogena semicolorata*), as well as to determine the presence of the thirteen species listed in the literature which were not confirmed in this study (*Ametropus fragilis*, *Ameletus inopinatus*, *Metreletus balcanicus*, *Siphlonurus armatus*, *Brachycercus harrisellus*, *Ephemera glaucops*, *Palingenia longicauda*, *Ephoron virgo*, *Choroterpes picteti*, *Ecdyonurus aurantiacus*, *Ecdyonurus siveci*, *Kageronia fuscogrisea* and *Rhithrogena germanica*). The rare or unconfirmed presence of most of these species is likely due to the lack of seasonal sampling. It is possible that they were present at some sampling sites included in this study, but at a very young instar or even egg stage, and as such were overlooked. Additionally, some species might have become extinct from the Croatian rivers, such as *Palingenia longicauda*, which at present likely only inhabits the Danube River and Tisza River in Hungary, Slovakia and Ukraine (Bauernfeind and Soldán 2012).

The Black Sea basin includes 62% of Croatian rivers (Jelić et al. 2008), which likely explains the higher number of mayfly species recorded in this basin than in the Adriatic Sea basin.

The Dinaric region is considered to be a biodiversity hotspot (Bãnãrescu 2004, Griffiths et al. 2004, Ivković and Plant 2015). Despite a similar number of taxa recorded in each ecoregion, the highest species diversity was recorded for the fast flowing streams and rivers in the Dinaric western Balkan ecoregion. Similar results were obtained in the study of aquatic dance flies in Croatia (Ivković et al. 2013). The lowest number of mayfly species was found in springs and lakes (Table 4). Various studies have shown that mayfly species diversity is generally low in spring areas (Berner and Pescador 1988, Bauernfeind and Moog 2000, Maiolini et al. 2011). The only spring with four species was the spring of the Ruda River (146) in southern Croatia (Fig. 1), which is largely fed with water from the Buško Blato reservoir (Štambuk-Giljanović 2001, Bonacci and Roje-Bonacci 2003) that is relatively rich in nutrients and organic matter (Štambuk-Giljanović 2001). Thus, mayfly communities in the Ruda River spring are more species diverse and have a high proportion of detritivores (Vilenica unpubl. results). Most mayfly species prefer lotic habitats with a larger array of microhabitats, and these are less diverse in spring areas and lentic habitats. The present study confirmed the results of many previous studies (Berner and Pescador 1988, Elliott et al. 1988, Bauernfeind and Humpesch 2001, Bauernfeind and Soldán 2012).

Mayfly larvae inhabit flowing and standing freshwater ecosystems where they occupy a range of microhabitats in correlation with different biotic and abiotic factors. Additionally, in running water habitats, due to the longitudinal gradient of the physico-chemical characteristics of the water, different parts of the watercourse are inhabited by different mayfly species (Elliott et al. 1988, Bauernfeind and Humpesch 2001). Cluster analysis (Fig. 2) based on mayfly assemblage generally showed that sampling sites are structured first by ecoregion and then by habitat type. For this reason, due to their morphology and water properties (Lucić et al. 2015), the large, slow Pannonian lowland rivers (Sava, Drava, Kupa) are separated from the other sampling sites situated in the Dinaric western Balkan ecoregion. SIMPER analysis (Table 5) showed that the Pannonian mayfly community consisted of species that prefer epipotamalic sections of rivers, such as *Caenis macrura*, *Procloeon bifidum*, *Heptagenia sulphurea* and *Potamanthus luteus* (Buffagni et al. 2007, 2009, Bauernfeind and Soldán 2012). Due to the two common mayfly species present in high numbers, *Baetis rhodani* and *Rhithrogena braaschi* (Vilenica et al. 2014, Vilenica unpubl. results), the investigated springs clustered together with the small mountain karst rivers. Larger karst rivers clustered together due to the presence of species with a wide ecological range as *Baetis rhodani*, *Centroptilum luteolum*, *Serratella ignita* and *Paraleptophlebia submarginata*, and species with a southern European distribution such as *Rhithrogena braaschi*. Another common species was *Baetis liebenauae*, previously recorded in smaller streams with a sandy or stony bottom as well as in large lowland rivers, where it can be found as a habitat specialist on macrophytes (Buffagni et al. 2007, 2009, Bauernfeind and Soldán 2012). The presence of a stony bottom and submerged vegetation may be a suitable habitat combination for the species. Further research is required to determine the more specific preferences at the microhabitat scale and physico-chemical properties of the water. The mayfly species diversity is generally quite poor in lentic habitats, though certain taxa can be very abundant. The main reason why lakes clustered together and apart from other sites was due to their species composition consisting of taxa from lentic (e.g. *Caenis horaria*) or a wide range of habitat type preferences (e.g. *Centroptilum luteolum*, *Ephemera danica*; Bauernfeind and Soldán 2012). Due to the presence and abundance of the species *Baetis rhodani*, Baetis cf. nubecularis, *Centroptilum luteolum*, *Serratella ignita*, *Ephemera danica* and *Paraleptophlebia submarginata*, the lower streams in the Plitvice Lakes National Park (sites 84 and 85) grouped together with the tufa-barriers (see also in Vilenica et al. 2014).

In comparison with the neighbouring countries and with consideration of their surface areas, the Ephemeroptera diversity in Croatia could be characterised as relatively high. Together with Croatia, Bosnia and Herzegovina is also situated in Dinaric western Balkan ecoregion (ER5) (Illies 1978). However, as its mayfly fauna is currently poorly known, with only 52 species recorded, and as a large part of Croatian territory belongs to the Pannonian lowland ecoregion, to which most of the Hungarian territory also belongs, the Croatian mayfly fauna was found to be most similar to the Hungarian fauna (75%, Table 3). This is due to the presence of widely distributed species and of the species inhabiting the larger rivers. Even though the mayfly fauna of Bosnia and Herzegovina is currently poorly known, 65% of the species were similar to the Croatian fauna. Thus, it is possible that a much greater similarity between these countries can be expected in the future. Italy is divided into two completely different ecoregions than Croatia: Italy (ER3) and Alps (ER4) (Illies 1978). It had a much higher mayfly diversity and the lowest similarity with the Croatian mayfly assemblage (55%, Table 3). This is possibly due to its geographical position and large surface area that includes a great variety of geographical features and diverse habitats. For example, the Alps, which are not present in Croatia, are well-known for their mayfly diversity and endemism, especially in the genus *Rhithrogena* Eaton, 1881 (Vuataz et al. 2011).

## Conclusions

As expected, this study revealed a higher number of mayfly taxa inhabiting Croatian freshwater habitats than known from the previous literature. As two of the most similar mayfly assemblages of the neighbouring countries have several taxa that could also inhabit Croatian habitats (e. g. *Baetis vardarensis* Ikonomov, 1962, *Rhithrogena picteti* Sowa, 1971, *Leptophlebia marginata* (Linnaeus, 1767), *Ephemerella notata* Eaton, 1887, *Caenis luctuosa* (Burmeister, 1839)) but were not yet recorded, due to the lack of systematic sampling in all seasons, future studies should include seasonal sampling of a higher number of sites and habitat types. Additionally, the main focus should be on the eastern lowland part of the country, where a lower number of sites was visited during this study.

In the present study, some interesting taxa with restricted European and local distributions were recorded (e.g. Rhithrogena gr. diaphana, Baetis cf. nubecularis and Ephemera cf. parnassiana). Considering these species were recorded from a small number of sites in this study, they could be considered rare. Future studies on the taxonomic status, ecological features and detailed distribution of these species is necessary.

Additionally, as *Baetis liebenauae* was recorded on larger karstic rivers, a different habitat type than previously known, more detailed information on its preferences at the microhabitat scale and water physico-chemical properties should be investigated.
